# From ‘safe haven’ to ‘zone of precarity’: locating Istanbul through the perceptions and everyday urban practices of skilled migrants

**DOI:** 10.1186/s40878-025-00432-4

**Published:** 2025-02-25

**Authors:** Ezgi Tuncer

**Affiliations:** Department of Architecture, Kadir Has University, Cibali Mah. Kadir Has Cad. 34083 Fatih, Istanbul, Turkiye Turkey

**Keywords:** Skilled migration, Privilege, Spatial precarity, Practices of everyday life, Istanbul, Urban safety, Discrimination, Ethno-spatial methodology, Online subjective mapping

## Abstract

This article seeks to position Istanbul through the practices of everyday life of middle-class, skilled migrants from both the Global North and South and their perceptions of urban safety and precarity. It examines individuals’ processes of migration to Turkey, revealing their initial impressions of Istanbul as a safe city of opportunities, and then analyses their everyday urban lives, highlighting hidden forms of precarity and discrimination. Through in-depth interviews with 45 women and 34 men—more than half of whom are North American and European—and participant observation in people’s living environments and at various social events, I argue that Istanbul, while perceived as a ‘safe haven’ at first, becomes a ‘zone of precarity’ where most of the participants have experienced intersectional forms of precarity, latent patterns of discrimination, and insecurities that belie the common perception that skilled migrants are privileged. To substantiate this argument, this ethno-spatial study presents an analysis of qualitative data as well as an online subjective mapping of Istanbul, where perceptions of urban safety and spatial precarity are displayed through socio-spatial experiences encountered in neighbourhoods, workplaces, and public spaces.

## Introduction

Following the 2018 economic crisis, Turkey, which was considered an upper-middle-income country (OECD 2024) even after the runaway inflation caused by the sudden collapse of the Turkish lira in November 2021 (Orhangazi & Yeldan, 2021), became one of the most affordable countries in terms of quality of life for skilled migrants from the Global North. Currently, this is particularly the case for those who earn foreign currency by working remotely or in Istanbul, where a better work-life balance can be achieved on a low budget. In line with Turkey’s safe-haven foreign policy (Oztig 2021), in which it has turned towards the Middle East (Aras and Karakaya Polat 2007), Turkey has recently been characterised as an ‘easy-to-enter, democratic, safe haven’ at the intersection of the Global South and North^1^(Solarz, 2020) for skilled people who have been forced to flee their countries due to conflicts and crises such as Russia’s invasion of Ukraine, anti-government protests in Iran, repression and fear in Afghanistan, and the devastating port explosion in Beirut, Lebanon. However, I argue that these skilled migrants, while mostly considered privileged (Fechter & Walsh, 2010; Favell et al., 2007; Waters & Brooks, 2011; Beaverstock, 2002; Sklair, 2000), share ‘the common status of being noncitizens with their underprivileged counterparts’ (Koh, 2020) and are seen as ‘flexible labour like low-skilled migrants’ (Zhan & Zhou, 2020). Moreover, they face inequalities and discrimination based on their ethnicity, nationality, and gender and experience different social class positionings and hierarchical stratification in Istanbul according to their origin and identity (Tuncer, 2024) due to the socially constructed perception of ‘skilled’ (Liu-Farrer et al., 2021; Bailey & Mulder, 2017) and skills recognition during modes of entry in the host county (Raghuram, 2021).

In this article, I claim that Istanbul, a dynamic global city where the vast majority of migrants to Turkey prefer to live due to its job opportunities and location, is perceived as a ‘safe haven’ at first, yet transitions into a ‘zone of precarity’ for some, where intersectional forms of precarity and discriminatory patterns are common. Aiming to situate Istanbul beyond the geographical and culturally conventional dichotomies, I delve into the everyday urban routines of middle-class, skilled migrants from both the Global North and South living in Istanbul in order to explore their perceptions and experiences of urban safety and precarity. My aim is to reveal that the highly educated middle-class is increasingly deprived of a secure urban life, and conversely, experience temporariness, unpredictability, and precariousness as a common factor of their lives.

The perception of safety addressed in urban studies has often been described in terms of fear, or as ‘the goal that can be achieved by the absence of fear’ (Cranston & Lloyd, 2019). For the most part, rather than understanding how a sense of safety develops, focus has been on what kind of fears affect people depending on variables such as race, nationality, ethnicity, class, gender, and age (Pain, 2001) and how these fears determine or restrict people’s use of urban and public space (Kern, 2005). Most of the geographical studies focusing on fear deal more specifically with the spatiality of crime and the feminisation of fear (Valentine, 1989), fear of the Other and the stranger (Lemanski, 2004; Panelli et al., 2005; Pain, 2009), and terrorism (Graham, 2006). Some studies argue that feeling safe in the city is related to familiarity (Fileborn 2016), being with others like oneself (Fechter, 2007; Yeoh & Willis, 2005), and creating ‘safe spaces’ (Roestone Collective, 2014) free from ethnic discrimination, racism, and sexual harassment. The phenomena of social ghettoisation (Fechter, 2007), spatial segregation, the formation of gated communities (Genis, 2007), and the spread of security technologies in daily life (Coaffee, 2016) are explained by the need for safety.

Rather than looking at the opposing dichotomy of safety and fear, I focus on the transitional space between the two and refer to this liminal space and condition as the ‘zone of precarity’. The first section presents a theoretical discussion of different forms of precarity and briefly explains the reason for focusing on practices of everyday life in order to understand the intersectional forms of precarity. The second section presents the ethno-spatial methodology, which is a combination of online mapping and ethnographic fieldwork, and includes a clarification of the utility of this spatial tool as a type of ‘counter-mapping’ within digital humanities. The third section discusses how different nationalities and professionals have perceived Istanbul as a safe haven and a city of opportunities. The final section is an interrogation of why and how the city has turned into zone of precarity for some. It also emphasises how the intersecting forms of precarity, discrimination, and insecurities create a stratification based on ethnicity, gender, and class.

### **‘Sorry**,** you’re a foreigner!’: Zone of precarity**

The precariat (Standing, 2011), in its most common sense, describes the social class experiencing economic precarity with a focus on employment and the labour market. In other words, this new concept, derived from the combination of the words ‘precarious’ and ‘proletarian’, can be defined as the deprivation of basic securities by workers. It was earlier used by Bourdieu to distinguish temporary workers from permanent ones, but was later popularised in the protests against austerity and economic insecurity in Europe in the 2000s (Paret & Gleeson, 2016). Precarity began to take hold more widely following the 1970s, when globalisation trends driven by the neoliberal market commodified workers and pushed them out of the guaranteed labour regime (Paret & Gleeson, 2016). Standing (2011), however, notably emphasises the gig-economy that emerged in the Global North, particularly in the US, after the 2008 economic crisis, and the transformation of flexible working practices that seem to liberate, yet more often result in the commodification of labour and the depletion of leisure time. Today, although there are still sectors and job opportunities in which basic labour guarantees such as adequate opportunities, protection from dismissal, and stable income still exist, labour markets and employment have also expanded in favour of using precarious labour, including the middle classes. In particular, the precariat is disproportionally led by women, youth, the elderly, the disabled, and migrants, who work in temporary, indefinite jobs with limited benefits, for short periods of time, with low wages per job, and become vulnerable to exploitation (Standing, 2011).

Neoliberal econo-politics’s precarisation of labour leads to increasingly uncertain employment prospects and unpredictability (Paret & Gleeson, 2016) becoming the norm. Although precarity is directly explained by economic insecurity, this ontological experience in fact rather points to an ambiguous way of being (Agergaard & Ungruhe, 2016) and permanent temporariness (Chacko & Price, 2021) that leave the individual in an indistinct space. In this sense, Standing (2011) draws attention to the exploitation and vulnerability of temporary, low-skilled migrant workers in particular in the formation of a precarious labour class through current migration policies. The two most prominent forms of vulnerability for migrants are illegality and deportability (Paret & Gleeson, 2016), that is, legal status or objective precariat (ILO, 2012). Even if it is assumed that this situation is inherent only to low-skilled migrants, while skilled migrants are predominantly seen as a cosmopolitan class with the privilege of mobility, the latter too are vulnerable to uncertainty, especially when working on temporary visas (González, 2022). In other words, to be skilled does not mean to be free from precarity. On the contrary, for skilled migrants, the majority of whom are middle-class, privilege and precarity go hand in hand. The skilled are subjected to differentiated rights and are not considered to deserve the rights enjoyed by citizens (Koh, 2020). Regardless of educational or skill levels, those with ‘precarious talents’ also fall into the flexible labour cluster (Zhan & Zhou, 2020) and may find themselves in precarious employment, ‘gig-work’, or uncertainty in terms of tenure and income (Chacko & Price, 2021).

Turkey currently recognises the rights of skilled professionals to work legally, such as temporary, indefinite, independent permits and Turquoise Cards^2^ with a benefit of pension security, health insurance, and temporary residence permits provided through their employment. Less than half of our respondents, the majority of whom from the Global South, however, do not have work permits, which makes them vulnerable, insecure, and open to exploitation. Having migrated mostly due to economic collapse, war, conflicts, or political pressure in their home countries, it is a common conception that they are obliged to maintain their jobs in Istanbul and thus are perceived as cheap labour in Turkey. Ultimately, since their cultural capital (Bourdieu, 1986; Lan, 2011) is not recognised, migration results in downward mobility. For example, a Pakistani man with a higher education received in the UK working in the media, while waiting for a work permit and earning a decent salary, would not appear to be within the boundaries of economic precarity. However, when this state of waiting turns into a permanent situation, it deprived him of his work permit and pushed him into many aspects of precarity. Cases of hidden, implicit discrimination based on ethnicity appear as a silent form of precarity that is rather subjectively perceived, as it is difficult to prove explicitly.

Subjective or perceived (ILO, 2012) aspects of precarity particularly reveal themselves in the ordinary details of people’s everyday urban lives in Istanbul. Discriminatory patterns such as misogyny, xenophobia, and verbal or physical sexual harassment in workplaces, neighbourhoods, and public spaces of the city cause people to feel precarious and unsafe. Many find themselves caught between the secure position of being an ‘acceptable migrant’ (Tuncer, 2024) and feeling the need to return home. Therefore, this article defines this transitional gap as the ‘zone of precarity’ in which many skilled migrants oscillate between having basic securities with decent employment and a legal work permit and experiencing insecurities, uncertainty, and discrimination. Recognising that precarity is temporal and scalar (Chacko & Price, 2021), this study attempts to understand their struggles against precarity by reinterpreting the opposing and dual power structures that organise everyday life, or strategies and tactics as de Certeau refers to them (1984, 34). Practices of everyday life consist of political power struggles between ordinary people and those who govern them. In contrast to strategies, i.e. the series of decisions that all typologies of power exert over those they rule, people produce tactics, i.e. spontaneous, silent, non-confrontational, unorganised, temporary, creative forms of resistance. Thus, uncovering the power struggles in all areas of individuals’ urban daily lives helps to form a greater understanding of latent patterns of discrimination as well as silent and intersectional forms of precarity. In order to do so, this study utilised an ethno-spatial methodology, which is a combination of online mapping that provides spatial analysis along with the qualitative data collected through ethnographic fieldwork.

## Spatialised precarity: towards an Ethno-Spatial methodology and the nature of participants

This study examines the interplay between power dynamics and socio-spatial environments, elucidating how they mutually shape each other—a concept akin to ‘building a bridge between those possessed of the sociological imagination and those imbued with a spatial consciousness’ (Harvey, 1973, p. 23). This emphasis on the inseparability of space, society, and politics, which forms a complex interwoven fabric, echoes Henri Lefebvre’s seminal work (1991). Therefore, cities are not only a stage or a setting in which everyday life takes place but also active spaces that can either create subjective and experiential feelings of insecurity or, on the contrary, enable individuals to feel safe. Since migrants in general are often perceived as temporary ‘guests’ separate from citizens (Chacko & Price, 2021), who do not deserve to have ‘the right to the city’ (Lefebvre, 1996; Kofman, 2024), the conflict over who owns the city can turn into deliberate practices of exclusion and discrimination.

Therefore, this study proposes an ethno-spatial methodological tool to reveal how precarity becomes spatialised. In order to better understand migrants’ urban precarity, I analyse migrants’ use, mobility, and personal experiences of the city, shaped around their home, workplace and socialising practices. Hence, it combines qualitative analysis of empirical fieldwork based on 79 in-depth interviews conducted with 45 women and 34 men from June 2022 to July 2023. The participants were accessed through the researchers’ academic and social networks in addition to snowball sampling and selection according to their level of education, professions, and origins as well as participant observations in people’s living environments and at various social events, along with online mapping of Istanbul.^3^ Following the first two parts of the semi-structured interviews on the process and drivers of migration and experiences in their countries of origin, the third and fourth parts focused on daily experiences at home and work and everyday use of public space in Istanbul, which provided data for the subjective mapping of urban life.

**Fig. 1:**
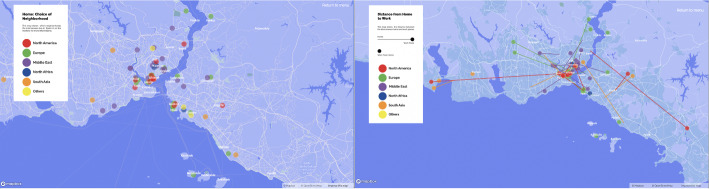
Maps of choice of neighbourhood and distance from home to work

The use of maps aids in the development of a theoretical understanding of everyday life through urban practices. De Certeau (1984, 91) interprets watching the city from a skyscraper as the gaze of the dominant power that distances itself from those walking on the street. Although looking at a city map similarly offers a panoptic perspective, the city maps of Istanbul resulting from this study also allow for a holistic approach while facilitating spatial analyses based on uses of the city that differ according to nationalities of skilled migrants. The first map (please see the website referred in the footnote 3), ‘*home and neighbourhood choices*’ (Fig. 1, on the left), reveals how skilled migrants seem to form clusters based on ethnicity and social class in certain neighbourhoods, thus creating socio-spatial segregation or hybrid spaces of various nationalities. Another, ‘*distances between home and work*’ (Fig. 1, on the right), gives a breakdown by country of origin and its relation to professions, locations of remote workers, and the relationship between choices of where to live and workplaces. The third map, ‘*social mobility in Istanbul*’, gives an idea about the locations of social and leisure activities of different nationalities.

**Fig. 2:**
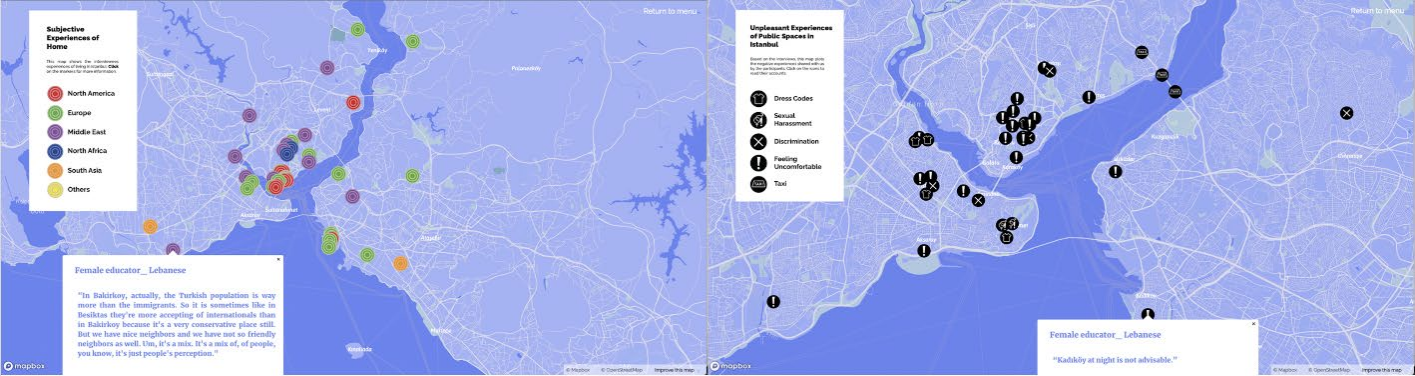
Maps of subjective experiences of home and safety in public spaces of Istanbul

Similar to ‘tours’ (de Certeau, 1984, 118), our map also provides information about participants’ individual experiences, subjective narratives, and lived space with a description of the city from below. Navigating through the maps of ‘*subjective experiences of home’* (Fig. 2, on the left), ‘*work experiences in Istanbul*’, and ‘*unpleasant experiences in public spaces in Istanbul*’ (Fig. 2, on the right) by clicking on markers and reading the narratives, shows migrants’ positive urban life experiences in conjunction with the insecurities and exclusionary and discriminatory practices that push them into the zone of precarity. In particular, the last map on urban safety depicts how some have experienced verbal/physical sexual harassment or cases of exclusion and discrimination. In addition, there are places where people do not feel safe and avoid going and also places where they have to protect themselves by developing dress codes for themselves. In this sense, the subjective mapping of urban life is a form of disclosure and counter-mapping (Drozdz, 2020; Kidd, 2019; Dalton and Stallmann 2018) as a form of resistance against strategies. It aims to reveal the invisible and offers insight into use of public space and everyday life activities in the city through documentation and analysis of anonymised experiences in accordance with ethical research practices. To create online maps with the free Mapbox service, related areas of the city are pinned according to the participants’ original responses from the in-depth interview process.

**Fig. 3:**
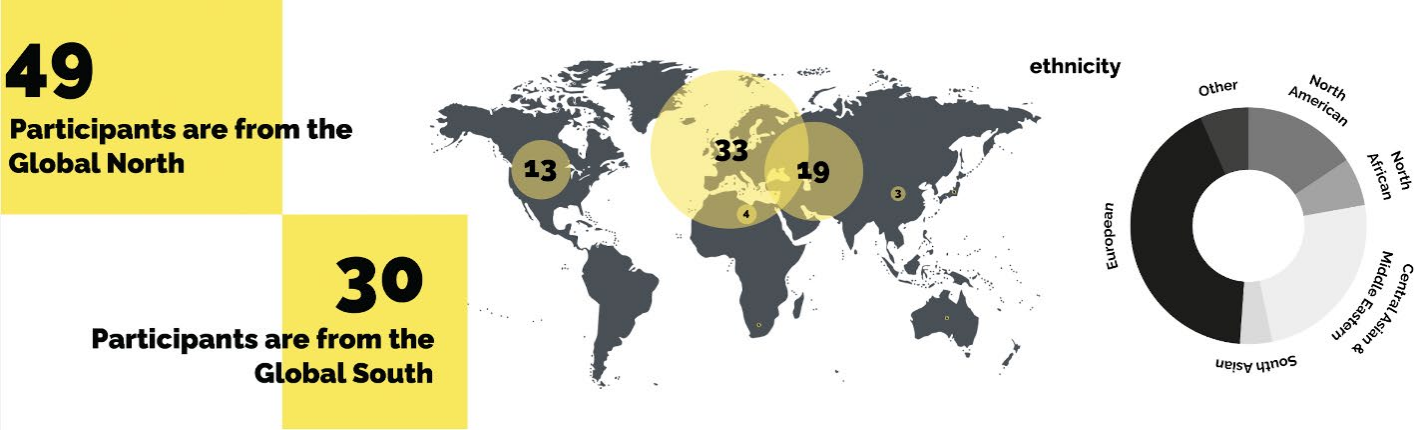
Participants’ origins

**Fig. 4:**
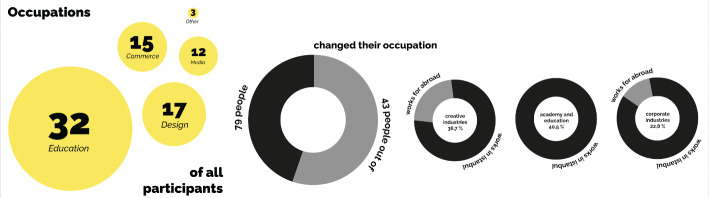
Participants’ occupations

**Fig. 5:**
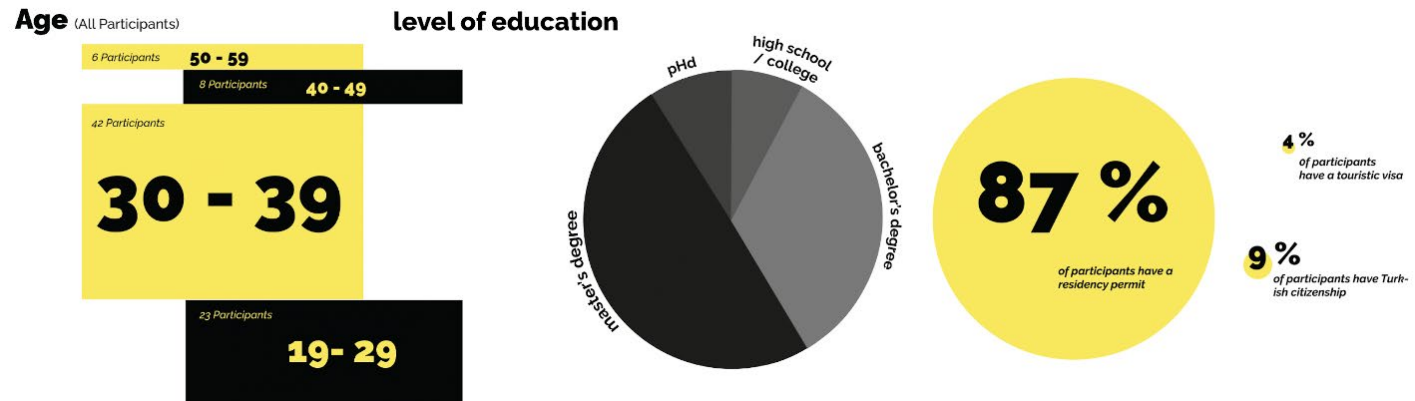
Participants’ age, level of education, and types of residence permits

Of the 79 participants, 49 from Australia, Canada, France, Greece, Ireland, Russia, the US, UK, and Ukraine, while the remaining 30 from Algeria, Azerbaijan, Bangladesh, Iran, Morocco, Uzbekistan, Pakistan, the Philippines, and Syria (Fig. 3). The majority of participants are fluent in English and work in the education sector (academics/lecturers at university or teachers at high schools or middle schools), while the rest work in art-design (architecture, industrial products, visual communication, design or fashion, music), business (administration, real estate, entrepreneurship), media (journalism, editing, translation, writing) (Fig. 4). While 36 participants had been able to maintain their previous occupation in Istanbul, others had to change careers. Some who work in creative industries and for corporations work remotely for foreign organisations or companies abroad and earn salaries in foreign currencies. About a quarter, who are in their 20s, are working university students and/or have started their first professional work experience in Istanbul, although more than half of the interviewees, who are in their 30s, are mostly senior in their respective careers (Fig. 5). About half of the participants hold a master’s degree and more than a quarter have bachelor’s degrees, while the rest, mostly academics, hold doctoral degrees.

Seven interviewees who are married to Turkish citizens have either been granted citizenship or hold family residence permits with a family visa. Although the majority of participants migrated alone, some settled in Istanbul with/for their Turkish spouses, and only nine of which have children, while 41 participants are unmarried. The majority of participants, however, are holders of short-term residence permits, with more than half holding work permits as well. One group of participants, mostly freelancers and remote workers, however, constantly travel into and out of the country on short-term, three-month tourist visas, continuing their remote work in other countries and then returning to Turkey since they use Istanbul as a base. The majority of interviewees have been living in Istanbul from five to 10 years, although a small number of them, especially those from Russia and Ukraine, settled in Istanbul in 2022. Thus, the majority of the interviewees speak Turkish only at a beginning or intermediate level, while around a quarter are fluent in Turkish, which includes those who have lived in Turkey for more than 10 years, those from Iran, and those with Turkish partners. A group of native English speakers, unrelated to the length of their stay, have either chosen not or felt no need to learn Turkish beyond the very basics (Fig.

**Fig. 6:**
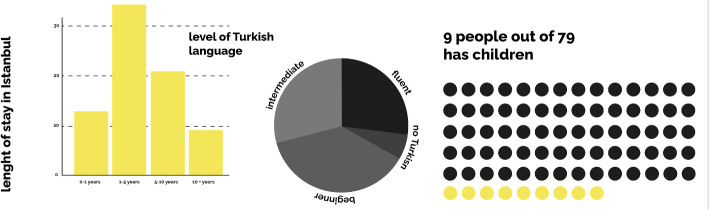
Length of stay in Istanbul, level of Turkish language, and number of people with children

6).

The two sections that follow the methodological approach of the research return to the main argument of the article. This includes a reading of the city from two different perspectives to exemplify how the city, which is first perceived as a safe haven for some skilled migrants, turns into a zone of precarity after a period of residence.

### Istanbul as a ‘safe haven’ and city of opportunities

Those working in the education sector in Istanbul, most of whom migrated to Turkey from North America and Europe, consider teaching at private schools or universities and gaining work experience in countries with growth potential, such as Turkey, as an important opportunity for professional career development. Even those with no previous teaching experience can find a job as an English or French teacher in Istanbul: ‘*I applied to all the French schools in Morocco*,* Algeria*,* Egypt*,* and Istanbul and they offered me a contract in Istanbul in June 2021. Istanbul seemed like a nice place to experience living abroad while teaching the French curriculum at the same time*’ (French, male, 32, July 2022). For many native English speaker, teaching English is seen as a guaranteed, well-paid, and secure position: ‘*I used to work for a company in Mecidiyeköy and I used to go to workplaces and teach English. They used to give me an envelope full of money*’ (American, male, 41, July 2022). As in the case of a female Bangladeshi teacher,^4^the way of life for some has become travelling from country to country.

Young academics who received their doctoral degrees, particularly from universities in the US, UK, and Canada, can find permanent positions without much difficulty, as they are highly sought after by Istanbul’s leading private universities. In addition, an American academic described the academic labour market in Turkey as a bit more balanced, less competitive, and more attractive than in the US. In addition to these perceptions, a lecturer’s marriage was also a reason for her migration: ‘*I was working as an architect in Chile. My husband is an academic from Turkey and it is almost impossible for him to find a job as an academic in the US. So*,* we said “Ok*,* why don’t we go to Turkey?*”’ (French, female, 39, July 2022).

Returning to Istanbul to work is another important reason for migration for the European, newly-graduated, young, middle-class who have received education in Turkey through Erasmus and similar exchange programmes and who enjoy living in Istanbul. Some interviewees also stated that they chose to stay in Istanbul after graduating due to the ease of finding employment. A teacher who had participated in the Erasmus programme expounded on the non-economic reasons that drove her to Turkey after graduation: ‘*In Europe there are already no job opportunities and the rents are quite high. I had a boyfriend at the time*,* he was Turkish…. I think it was the easiest option*’ (Spanish-French, female, 27, November 2022).

Some interviewees emphasised Istanbul as a convenient and favourable global market for starting a business, as its location allows for constant mobility: ‘*Istanbul is great for entertainment because you have Uludağ in winter. If you want a beach*,* you can get on a plane and go to Bodrum. There is Sapanca for camping on a low budget. Kadıköy is also great for my restaurant* (French, 33, male, December 2022). An accountant living in Istanbul for more than a year drew attention to the financial advantages of living in Turkey while working for a US-based company: ‘*My life was good in America. I had a good social life and family life*,* but I got bored. I came to Turkey. I earn… perfect money to live in Turkey. I can also work… and travel when I want*’ (American, female, 33, October 2022).

Similarly, the presence of transnational journalists who use Istanbul as a base to cover the conflicts and wars in neighbouring countries also points to an important group of skilled migrants. The city’s position as an important global station between Europe and the Middle East makes it easy for many journalists to travel to various neighbouring countries to report while residing in Istanbul: ‘*I am going to India next month*,* then Greece and I have direct flights everywhere. So*,* Istanbul is a very good location*’ (Swedish, male, 30, November 2022). A Canadian journalist stated that he had travelled to Iraq after the 9/11 attacks to cover the war on terror but settled in Istanbul because Turkey is a safe country close to all the areas he covered.

Opposition to Russia’s invasion of Ukraine as well as fleeing the country’s deteriorating economic situation and high levels of corruption are motivating factors for many to leave Russia. A fashion designer said that Russia’s invasion of Ukraine was the breaking point to leave the country: ‘*If you openly oppose this war and call it a war*,* you go to jail for years… So*,* we prepared all the papers very quickly and bought the tickets and came to Turkey*’ (Russian, female, 35, November 2022). Russia’s invasion of Ukraine has also led many Ukrainian citizens^5^ to migrate to Turkey and see it as a temporary refuge, as it remains one of the best options due to its visa-free regimen and geographical proximity. The closure of many services and difficulties in online communication following the outbreak of the war have slowed down the work of many and jeopardised their positions. As most of the Russian and Ukrainian participants work in sectors such as media, journalism, IT, social media, photography, and cinematography, they have found the chance to maintain a fairly high quality of life in Istanbul by working remotely.

Turkey’s cultural similarities with Middle Eastern and North African countries and its geographical proximity are one of the main reasons why many from those countries settled in Istanbul: ‘*I did my master’s and PhD in the UK. Then I wanted to be closer to my family*’ (Iranian, female, 39, July 2022). Most stated that they had either visited Turkey on holiday before or had met Turks in their home country, which had familiarised them with Turkish culture. Some interviewees, particularly from Syria and Pakistan, established an imaginary kinship through religion before migrating. Having learnt Turkish beforehand is another important driving force for those who foresee a medium/long-term stay in the country to migrate to Turkey.

In addition, the fact that legal entry into Turkey is much more accessible than for European countries and beyond and that it is easier for skilled people to naturalise are also among the reasons many come to Istanbul: *‘My naturalisation application was an easy procedure; it was only my fifth year here’* (Syrian, male, 28, June 2022). A Moroccan woman working as a real estate agent pointed to the presence of Turkish brands and chains in her country and the ease of entry provided by Turkey’s trade agreements and visa-free policy. A Pakistani man working as an engineer and seeking university opportunities abroad explained that he had to settle in Istanbul because many countries in the Global North had rejected his visa application, whereas Turkey has an institutionalised foreign policy plan for high school graduates from the Global South to pursue higher education within the Turkish university system. There are those, however, especially Central and South Asians, who see Turkey as a springboard between their home countries and possible careers in Europe or North America and study in Turkey with the dream of working and living in Europe: ‘*One of the reasons I came to Turkey was the standard of education. But now that I have found a job in London*,* I will move*’ (Afghan, male, 31, December 2022).

For many, achieving better opportunities and a secure life in a relatively stable, wealthy, liberal country is another major motivation to migrate. A researcher specifically mentioned women’s public presence in Istanbul and gender equality: ‘*There is more of a female culture in Turkey. I can say that women here live their lives as they want…. I am both surprised and happy that they are so dominant and independent*’ (Pakistani, female, 29, July 2022). A psychologist explained that being forced to cover or shave her head against her will was the most obvious reason that triggered her to leave Iran and move to Istanbul: ‘*Istanbul is more open-minded. It is much more modern*,* friendly*,* and accepting*’ (Iranian, female, 31, April 2023). Some interviewees explained that they had to leave their countries due to oppression and/or political and economic violence: ‘*Lebanon is not safe and not as stable as it used to be. The government and police cannot intervene in domestic violence. Tribes are powerful. Honour killings are common. LGBT people are not welcome in society and the work environment*’ (Lebanese, female, 22, April 2023). Moreover, a journalist and her husband wanted to marry after having a long, secret relationship in Iran but could not because of Iranian laws, so they decided to emigrate: ‘*In Iran*,* my husband and I were constantly facing censorship. Here I don’t have to wear a headscarf and this is very exciting for me; I can be myself! I had an abortion when I was young and I always thought I am not a good woman*,* I am a prostitute. I don’t deserve a good life*’ (Iranian, female, 38, September 2022).

## Urban experience of precarity and discrimination

### Labour spheres

Language teaching is perceived as an in-demand, well-paid, and secure job by most educated migrants in Istanbul. The hierarchy defined between native speakers and others in terms of their language capital (Lan, 2011), however, results in a wage gap and pushes some into precarious positions: ‘*Our school has a policy that if you are a foreign teacher*,* a designated teacher*,* you get paid in US dollars*’ (American, female, 52, June 2022). On the other hand, some native English-speaking teachers raised the issue of the lack of a work permit, which makes it illegal for them to work and/or stay in the country. Similarly, a British academic described her frustration with her employer forcing her and her colleagues to work overtime, especially during the Covid-19 pandemic, despite sacrificing her salary to achieve a work-life balance in Istanbul. She was also obliged to give private English-language classes as her salary has halved in foreign currency due to the depreciation of the Turkish lira. This pushed her and others into the zone of precarity: *‘The reason why I continue studying is this: journalism is very unstable because I am freelancing*’ (French, female, 37, June 2022). Despite being perceived as privileged ‘whites’, some female participants from the Global North reported that Turkish men see them as hypersexual and open to all kinds of sexual advances, which leads to frequent verbal sexual harassment in the workplace. One teacher related that she was harassed while running a language course: ‘*Since I am a Christian woman*,* they think I am a prostitute. I feel this from many men*’ (Greek, female, 32, June 2022). However, there are also men from the Global North who say that they experience attempts to belittle due to the fact they are foreigners: ‘*Since you’re a foreigner*,* lots of Turks say it’s impossible for you to understand Turkey*,* Turkish history*,* or Turkish culture since you’re not Turkish’* (American, male, 41, October 2022).

**Fig. 7:**
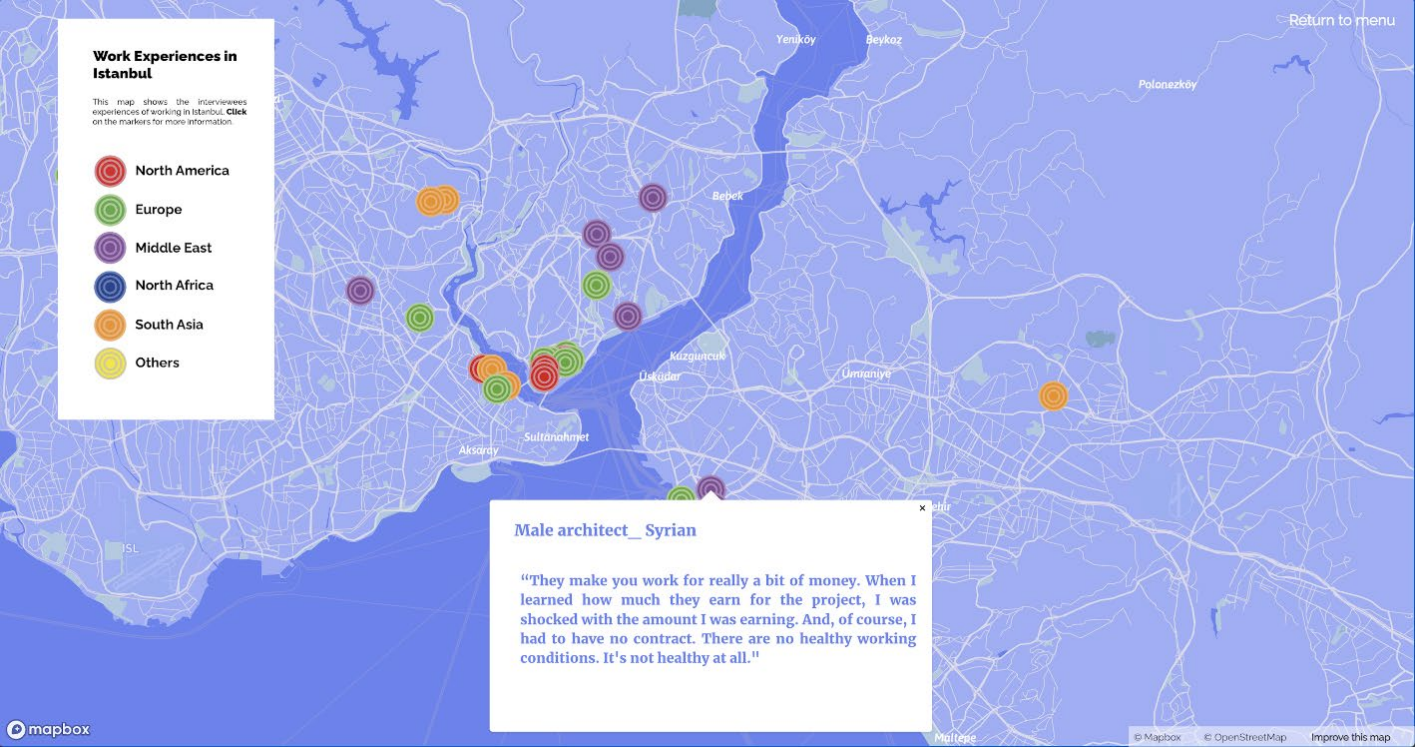
Syrian architect’s precarious experience of exploitation at work

Among the interviewees, Syrians and Iranians in particular had been asked to work without a work permit for a three-month trial period, during which they were paid low wages either in cash or from a special account. After the illegal probationary period, companies have to apply for work permits for their foreign employees, which they see as a burden, as it increases the gross wage the company has to pay the employee, and they often delay or protract the process: ‘*It was like this: “We have applied. I will tell you what documents you need…. We are waiting for feedback.” In the end*,* I realised they didn’t even apply in the first place*’ (Iranian, female, 32, June 2022). In addition, many men from the Global South described how they are not seen as equal to their colleagues from Turkey or the Global North, which presents the intersectionality of precarity (Fig. 7). An architect explained the challenges of being a skilled migrant from the Global South: ‘*There are two types of foreign workers: one is from European countries or the US and the other is from Asia or the Middle East. Even if you are very qualified… even if you have language*,* technical knowledge*,* communication*,* you get low wages*’ (Afghan, male, 31, December 2022).

There is also a significant class gap in terms of consumption, expenditure, savings, and standard of living between those earning salaries in foreign currency and those earning salaries in Turkish lira. Although the majority of interviewees earning foreign currency were from Global North countries such as the UK, US, and those in Western Europe, it does not translate into all Global Northerners being privileged, as on the contrary, some also have experienced economic precarity: ‘*I like the neighbourhood; it would be more fun if the economy was better and I could have the resources to really enjoy it. But at least there is a “beach” and I can ride a bike*,* it’s free*’ (American, male, 36, October 2022). This difference in social-class positioning naturally has determined their choices of neighbourhood and spatial mobility in Istanbul.

## Home spheres

**Fig. 8:**
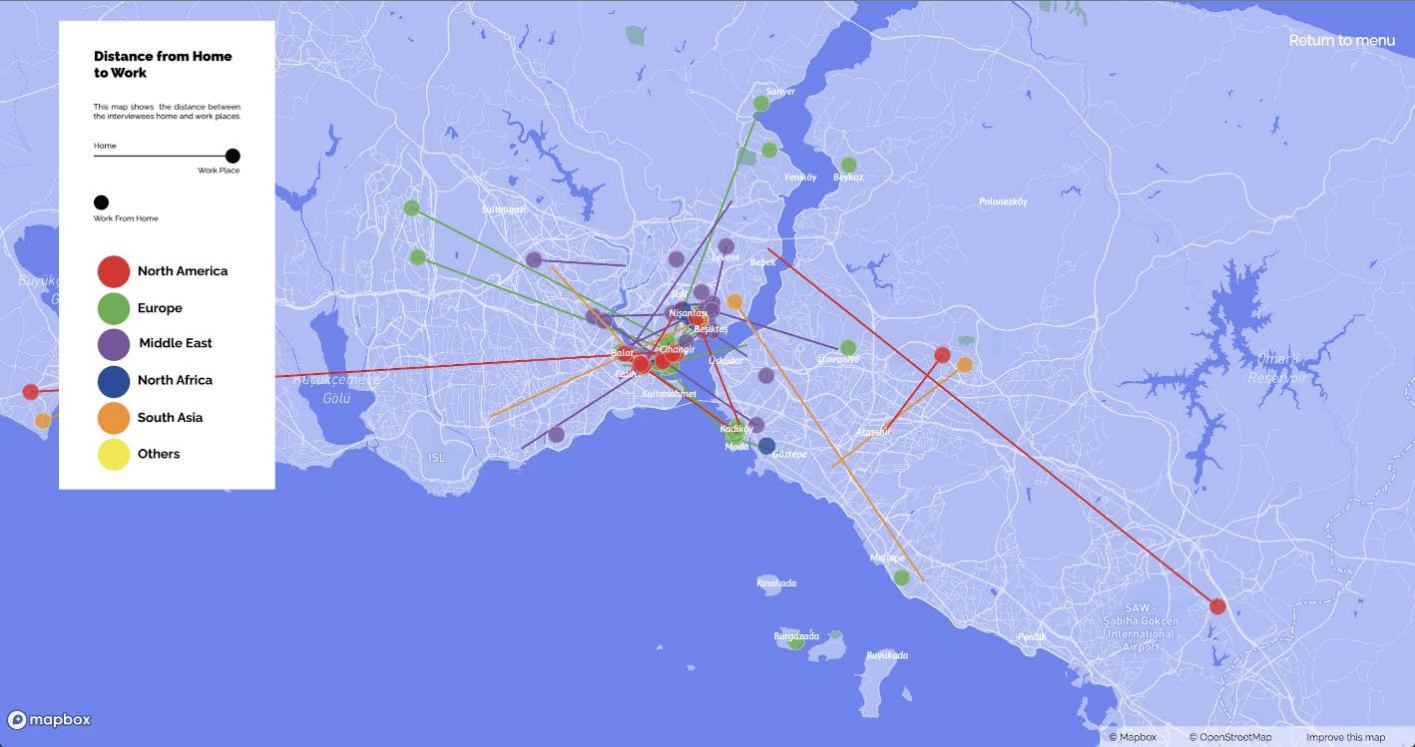
Map of distance from home to work

Some language teachers and academics working on campuses commute the farthest from the city centres (Fig. 8). They have to take company shuttles to their campuses in relatively peripheral areas of the city, a daily occurrence in Istanbul’s notoriously heavy traffic, which results in them having little time to socialise during their non-work hours of the workweek. However, as a result of the Covid-19 pandemic and increased opportunities to work from home, some of the European and American respondents living in Cihangir and Moda continued working from home either as freelancers or remotely for companies abroad, newspapers and news channels, and international NGOs.

While most of the earners of foreign currency earn enough to rent spacious apartments with Bosporus views in historic and central neighbourhoods with very high market value; benefit from the city’s vibrant and lively cultural life; enjoy high-quality food and beverages, entertainment, and sports facilities; budget for luxury consumption; and frequently holiday in Europe or within Turkey, those who earn Turkish lira have a much more limited social life. Ultimately, the majority of Americans and Europeans are concentrated in neighbourhoods such as Cihangir, Galata, Moda, Yeldeğirmeni, and Balat. The reason why most of them opt for these neighbourhoods is the presence of people like themselves, which offer ‘chosen families’ and a more secular, urban, European lifestyle. These neighbourhoods are also close to cultural and entertainment centres such as Taksim and Kadıköy, where transnational communities meet for various events and activities. This clustering, however, also brings with it the threat of transnational gentrification (Hayes & Zaban, 2020), which disturbs even foreigners who chose to live in certain neighbourhoods before the economic crisis since they liked the areas’ relatively calm and local production: ‘*We really like Kadıköy but five or six years ago it was less crowded and it was actually mostly Turkish people. But now you can see more foreigners*’ (Russian, female, 32, August 2022).

**Fig. 9:**
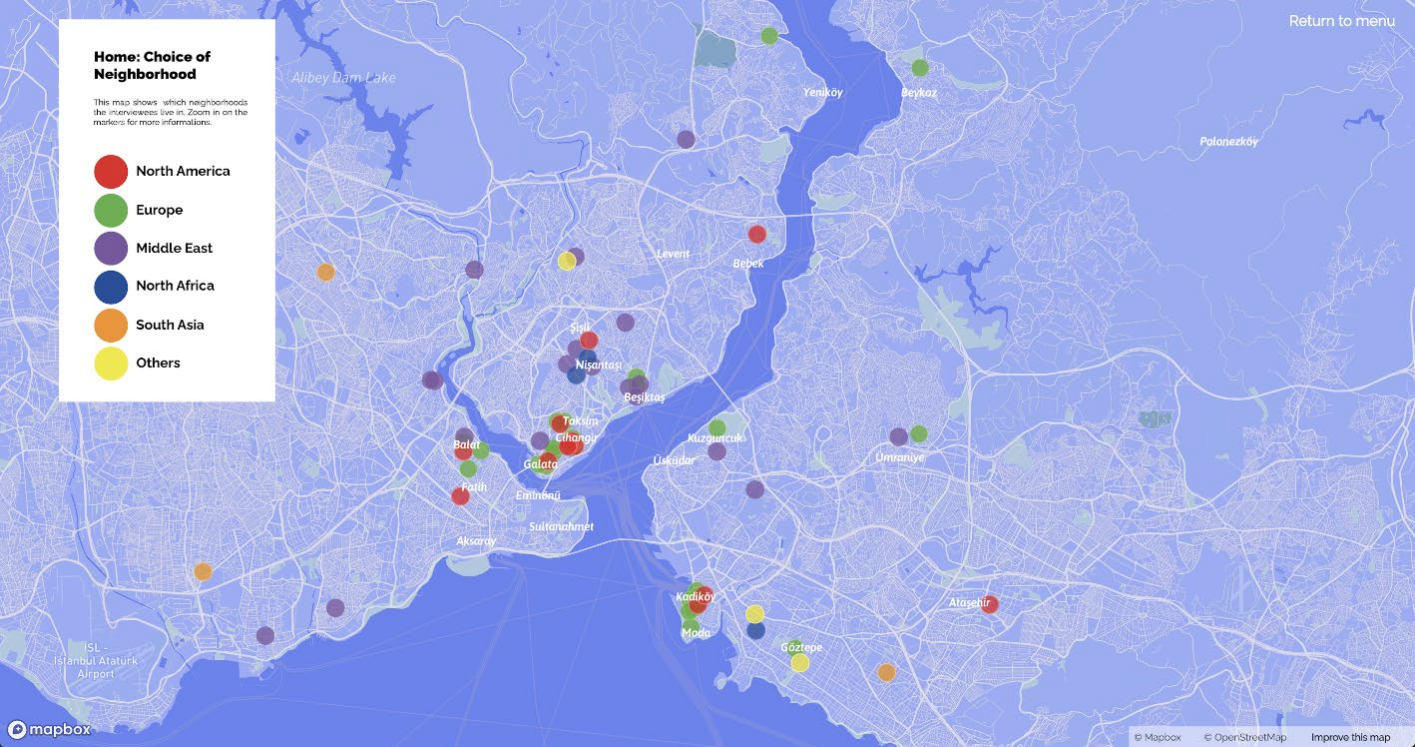
Map of neighbourhood choices

While Beşiktaş is a neighbourhood mostly preferred by a set of Europeans and partly Middle Easterners, the map in Fig. 9 reveals that Kurtuluş-Şişli is preferred by a more hybrid group of Middle Easterners, North Africans, and Americans. Some Middle Easterners and South Asians have opted for neighbourhoods such as Sultangazi, Esenyurt, and Kartal, which are relatively distant from historical centres, since they offer more economic living conditions or have diaspora networks. A small number of Americans and Europeans chose to live in the smaller neighbourhoods along the Bosporus and on Istanbul’s islands because they are quieter and are home to local, ethnic minority cultures.

**Fig. 10:**
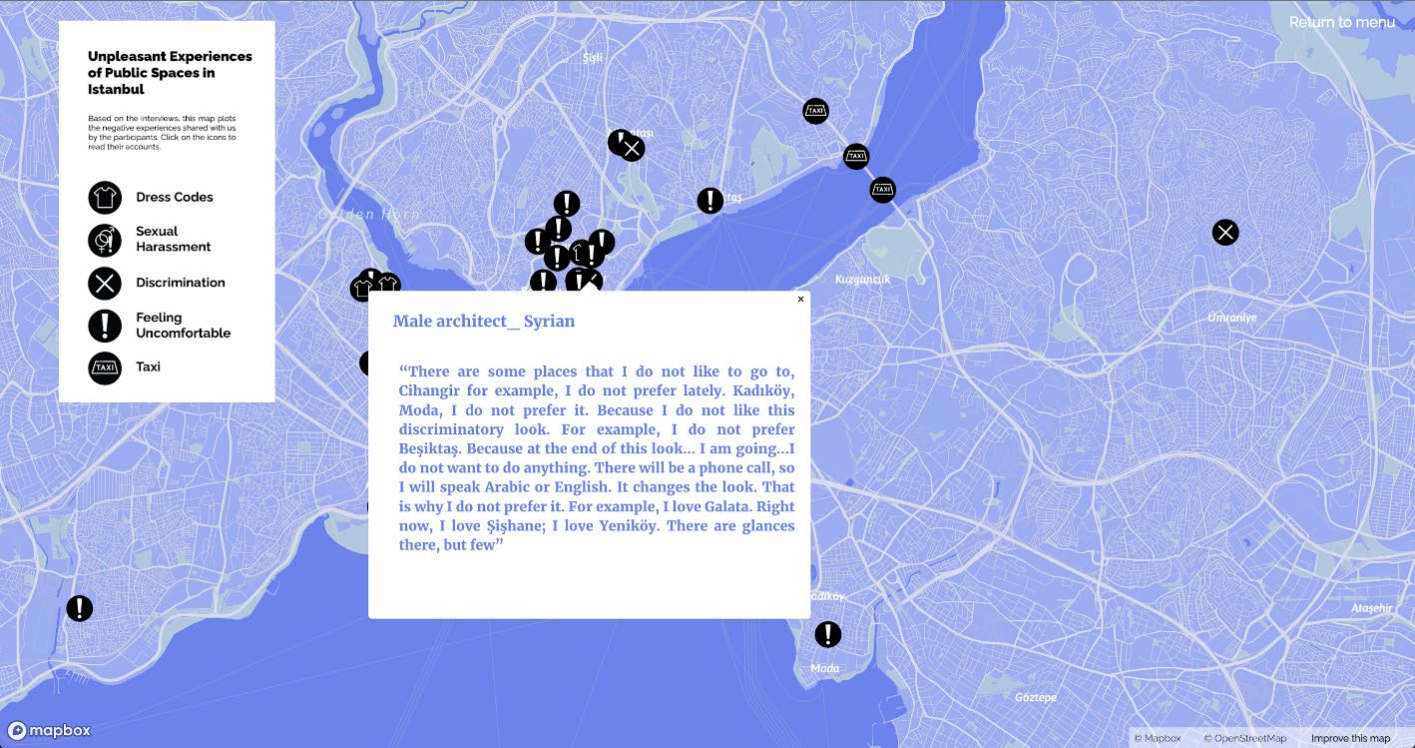
Ethnic discrimination/xenophobia in Istanbul as an intersectional form of precarity

Some interviewees reported experiencing discrimination in renting an apartment simply because they are foreigners: ‘*It was stressful and difficult to talk to real estate agents when looking for an apartment and sometimes they didn’t want to talk to you because you were foreign*’ (British, male, 27, June 2022). An architect who has been living in Turkey for more than 12 years described similar xenophobia: ‘*The landlord told me that he only wanted to give his house to a Turk. I told him I am a Turkish citizen and showed him my ID but he said*,* “No*,* you are not Turkish*”’ (Afghan, male, 31, December 2022). The recent, ongoing rent crisis has also put some interviewees in a difficult situation. Some landlords have not complied with the annual 25 per cent rent increase limit and have tried to raise the rent before the one-year lease period expires. Landlords also increase the dimension of bullying and intimidation by thinking that foreigners ‘*do not know the law*,* cannot get legal assistance*’ and ‘*are rich people who receive high salaries in foreign currency*.’ An editor explained his experience: ‘*My landlord said* “*I need my apartment for my grandson*,* so you need to move now.” She’s tried to increase the rent 100 per cent every year. When I told her there’s a legal limit*,* she said it doesn’t apply to me because I’m foreign*’ (American, male, 41, October 2022). The increased anti-Arab sentiment that has developed in certain neighbourhoods in Istanbul has affected many darker-skinned foreigners. Speaking Arabic on the street, on public transportation, and in taxis can often result in problems from locals (Fig. 10). Although Syrians constitute the majority of those subjected to discrimination, locals refer to anyone who speaks Arabic, Persian, or Urdu with the homogenous epithet of ‘Arab’: ‘*I was speaking Arabic with two of my friends. A Turkish woman turned and started shouting. She was saying*,* “Arab*,* Arab!” I said*,* “What does it mean to be Arab?” She said*,* “I will call the police!” I said*,* “Call them. I have an ID card*,* so why should I be afraid?”’* (Palestinian, female, 22, April 2023).

## Leisure spheres

**Fig. 11:**
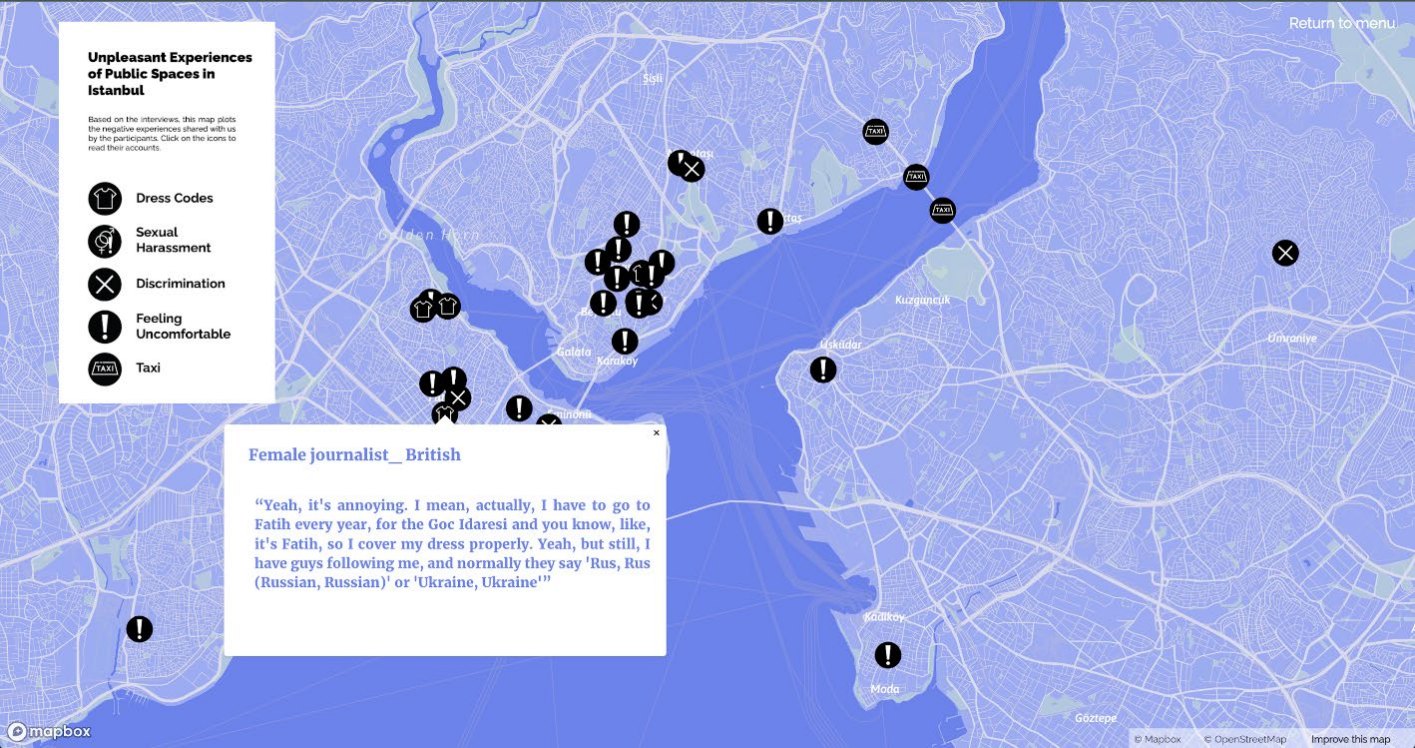
Social restrictions, dress code, and feeling unsafe in specific neighbourhoods of Istanbul

Female interviewees referred to Fatih as the most unsafe neighbourhood, which they avoid due to its conservative Islamic character that dominate the public sphere where women are exposed to the gaze and repudiations of religious men and even verbal sexual harassment from them because of their clothing and behaviour (Fig. 11). Although some of the women have not experienced danger themselves, due to the collective discourse passed on from other women, most avoid the Fatih-Eminönü area and the old city/historical peninsula. In Tarlabaşı (historical, ethnic minority, undergoing gentrification), which ranked second on the list of unsafe neighbourhoods, feelings of insecurity were fed not only by masculine, oppressive, and conservative spatial violence but also by xenophobia and a reputation for theft.

On the other hand, the majority of people from the Global North were aware that there is crime and danger in Istanbul, but still argue that the city is still much safer than cities in ‘Western’ countries: *‘In my old neighbourhood [in Washington*,* D.C.] there were shootings and stabbings every day. I’m not saying it doesn’t happen in Turkey*,* but it’s not as visible*’ (American, female, 52, June 2022). Most people from the Global North explained that they feel comfortable walking alone on the street at night and while using public transportation in Istanbul. Some of the white foreign men interviewed, however, have experienced how some Turkish men see them as less masculine since they are possibly uncircumcised in competition with themselves: *‘A street watchman stopped us to check our IDs and tried to insult and intimidate me in front of my Turkish partner. They think I’m stealing “their women” and have this inferiority complex about it*’ (American, male, 41, July 2022).

**Fig. 12:**
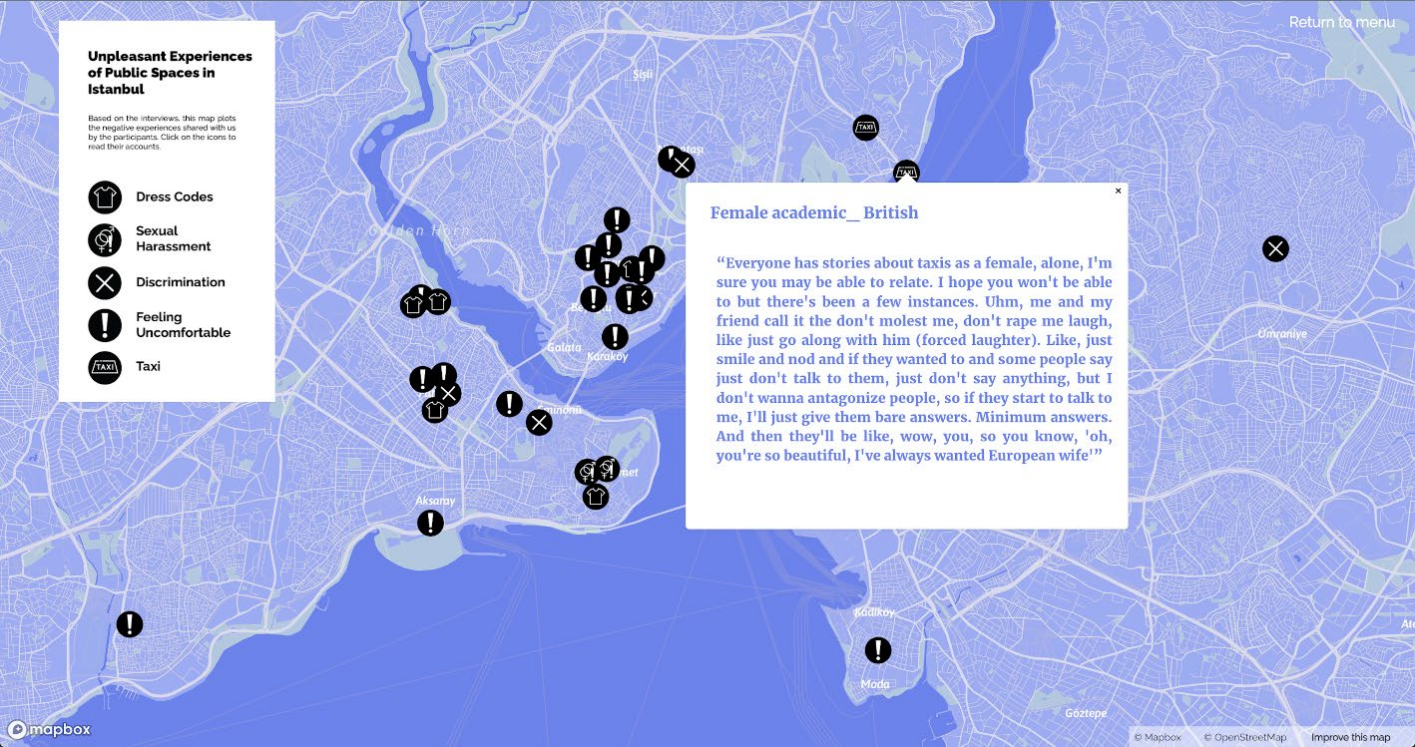
Experiences of sexual harassment in taxis in Istanbul

According to the female and LGBTQI + interviewees who have been subjected to aggression and sexual harassment in public spaces, taxi drivers were particularly bad offenders (Fig. 12). A teacher from Azerbaijan explained that taxi drivers have realised that he is gay and verbally harassed him and have also suggested he perform sex acts for them during the ride.

A female Iranian software engineer explained that after experiencing several incidents of men rubbing their genitals against the backs or sides of women on crowded metrobuses or quickly touching their buttocks as they pass by, she has learnt to be more cautious and to react. A teacher also described her perception of Turkish men being more daring, especially towards foreign women: ‘*In public transport*,* sometimes I vaguely feel the hands of some older men. They want to touch me by making it look like an accident. They cannot do this easily to a Turkish woman. They can recognise that you are a foreigner*’ (Greek, female, 32, June 2022). Similarly, an American researcher related how in historical and touristy neighbourhoods such as Sultanahmet, the way Turkish male shopkeepers’ approach and talk to foreign women often includes verbal sexual harassment.

## Conclusion

This study firstly set out to understand why skilled, middle-class migrants from the Global North and South choose to live in Istanbul and how they perceive and position the city. It presents a holistic analysis of their daily urban lives in their workplaces, neighbourhoods, and public spaces, revealing cases of discrimination, legal and subjective forms of precarity, and spatial experiences of unsafety and insecurity. Ultimately, the ‘dynamic’ and ‘dazzling’ global city of Istanbul is perceived as a safe haven for many reasons upon first glance, yet for some, the city turns into a zone of precarity in one way or another from time to time for most, with various and prevalent forms of vulnerability and discriminatory patterns. Therefore, beyond traditional geographical and cultural dichotomies and common perceptions, Istanbul is located in a ‘third space’ (Soja 1999): A hybrid city that belongs neither to the Global North nor Global South, illustrating the problems of this dichotomy. Turkey is neither a developed nor undeveloped country, neither in Europe nor the Middle East, neither fully democratic nor fully repressive, neither totally religious nor totally secular, neither inclusive nor exclusive, neither safe nor dangerous.

People from both the Global North and South migrate as an individual tactic to escape either the economic precarity in which they are trapped or political, religious, and social pressures, i.e. the strategies of various forms of power in de Certeau’s terms. For many of our interviewees from the Middle East, North Africa, and South Asia, Istanbul’s geographical proximity allows them to visit their families in their home countries frequently while Turkey’s socio-cultural similarities make them feel safe. Ease of entry, access to citizenship, and the opportunity to attain university degrees along with their perception of Turkey as a stepping stone on the road to Europe are important reasons to migrate to a ‘freer, more prosperous, and developed’ country, and more specifically to the global city of Istanbul. For people from the Global North, on the other hand, migration with the intention of finding a permanent job, developing a new career, establishing a healthy work-life balance, and obtaining a ‘higher quality of life at a cheaper price’ are critical reasons they settle in Istanbul, as it is a convenient nodal point between Europe and the Middle East, with Turkey being home to both cultures. All of these reasons show that the interviewees’ perceptions of Turkey and Istanbul are interwoven with ideas of safety. Whether they are fleeing precarity or oppression, Istanbul was perceived as a safe haven and a good or advantageous option by all participants.

This qualitative analysis of the practices of everyday life, however, reveals that apart from the most privileged group of foreign currency earners—entrepreneurs, international journalists, remote workers, and in part educators—middle-class, skilled migrants are subjected to intersectional forms of precarity, insecurity, and/or discrimination, even those who have to endure inadequate opportunities and the conservative cultural character of the country. In the workplace, those from the Global South are undervalued and exploited as cheap labour compared to both their local and Global Northern colleagues. Many face ethnic discrimination beyond xenophobia in certain neighbourhoods, on the street, on public transport, and in taxis. Thus, while fleeing war and conflict, political and religious oppression, gender inequalities, and homophobic violence in their home countries and imagining Turkey as a safe haven, they are caught in a zone of precarity in Istanbul defined by patterns of discrimination and legal insufficiencies. Some Global Northerners are undoubtedly more privileged in terms of being able to use their language capital, are positioned higher in hierarchical structures based on identity and origin (Tuncer, 2024), and have access to remote employment for international companies and institutions thus earning foreign currency-indexed income. Given this position, however, that not experience the same conditions in Istanbul. While some Americans and Europeans flee the hyper-capitalism, precarity, unemployment, and expensive and difficult living conditions in their home countries, they also fall into the zone of precarity in the perceived safe haven of Turkey. Some of them have no choice other than earning Turkish lira, freelancing, and/or taking on several jobs at the same time and are at times subjected to xenophobia and exclusion.

While it is men from the Global South who are most frequently excluded and experience xenophobia and verbally harassment in public spaces in Istanbul based on language and skin colour, it is women and LGBTQI + of all national origins who are most frequently subjected to verbal and physical sexual harassment, homophobia, misogyny, patriarchal structures, and masculine domination of public space. Particularly in this sense, this urban study reveals that precarity is also spatial by presenting the cases of discrimination and sexual harassment faced by migrants in public spaces through subjective mapping. Thus, it also goes beyond illustrating only economic and objective precarity by focusing on the subjective, individual, and ordinary details of urban life. The ethno-spatial methodology, based on the premise that the social is also spatial, reveals that the subjective forms of precarity experienced by individuals are also related to their experiences of home, local working practices and environments, and their capacity to use the city; in other words, precarity is directly spatial.

Finally, I would like to conclude this article with a few suggestions to facilitate the participation and integration of skilled migrants into urban and working life in Istanbul and to reinforce their perception of urban security. First of all, residence permits with different statuses should be defined for highly educated migrants. The ambiguity and lack of information in residence permit applications should be eliminated; the government should provide interpreters at migration offices; in-person appointments should be held at the neighbourhood level instead of at a few noncentral locations. The government should provide clear, precise, and correct information and legal advice concerning residence/work permits. There should be an audit/complaint mechanism on the provision of work permits. The government should support business start-ups and individual entrepreneurship and their conditions should be reviewed. Secondly, the government should provide a legal service and counselling for the intricacies of housing rentals. Housing quotas, similar to those already in place, can be implemented to prevent clustering in certain neighbourhoods and ensure cultural diversity. Neighbourhood-based events such as food, music, games, and language exchanges can be organised to bring transnationals and locals together. Thirdly, the government should provide employers, shopkeepers, and government employees with training to diminish and counter racism and sexual harassment. Moreover, government campaigns to raise awareness of these issues should be conducted on public transport, in neighbourhoods, and across the media.

## Data Availability

The availability of the qualitative data obtained from the participants which support the findings of this study is subject to restrictions in accordance with scientific research ethical principles within the scope of Kadir Has University Scientific Research and Publication Ethics Directive [02.02.2022–24790]. Therefore, not all interview data can be shared with the public for confidentiality reasons. However, partial data shared to contribute to the advancement of scientific research, including the in-depth interview layout, an anonymised interview sample and the website of our project can be accessed at: .

## Footnotes

1 Many of the countries in Latin America, Africa, Asia, and Oceania that are considered less developed are referred to as countries of the Global South because of their lower levels of economic power, industrialisation, and income; greater socioeconomic inequality; and dependence on global economic systems controlled by the countries of the Global North. However, the Global North-South dichotomy points to a highly misleading, reductionist, polar opposition that homogenises both sides and misses out on the global trends, hybridity, and nuances that still perpetuate colonial domination. Although“Western” countries are considered to constitute ‘the centre’, currently, economic hubs such as China and India are also shaping a decolonial formation and breaking down the centre-periphery dichotomy. Therefore, in this article, while recognising the ambiguity and problems of these terms, the Global North and South binary had to be used for comparisons instead of using the names of each individual country in every mention. 

2 https://www.csgb.gov.tr/uigm/calisma-izni/izin-turleri/.

3 https://subjectivemapping.superhi.hosting/.

4 https://gdlm.superhi.hosting/fteacher.html.

5 https://gdlm.superhi.hosting/feditor.html.
